# Acceleration of shooting and bouncing ray method based on OptiX and normal vectors correction

**DOI:** 10.1371/journal.pone.0253743

**Published:** 2021-06-25

**Authors:** Gaogui Xu, Chunzhu Dong, Tao Zhao, Hongcheng Yin, Xuan Chen

**Affiliations:** 1 Science and Technology on Electromagnetic Scattering Laboratory, Beijing, China; 2 School of Information Engineering, Communication University of China, Beijing, China; Central State University & Ohio University, UNITED STATES

## Abstract

The present paper deals with a new efficient shooting and bouncing ray (SBR) method based on OptiX and normal vectors correction. The basic idea is to make full use of the computing resources of the RTX series graphics cards. For ray tracing, the algorithm uses OptiX to invoke the built-in RT Cores of hardware. Thus, a fast intersection test can be implemented. To reduce the error of ray tracing caused by the facetted surface characterizing the curved surface, the direction of the reflected ray is corrected by normal vectors correction. Additionally, multiple GPU cores are invoked to accelerate the calculation of far-field integration of millions of ray tubes, which can improve the efficiency of the algorithm while reducing the data transmission time of heterogeneous devices. Simulation results show that the ray path after normal vectors correction is consistent with the theoretical results, and the algorithm can predict the RCS of arbitrary facetted geometries, which is 60 times faster than the SBR method based on kd-tree.

## Introduction

Computational electromagnetic theory has received more attention from researchers and engineers over the last decade. This fact is due to their wide applications such as antenna design and analysis [1, 2], stealth technology [3], and radar cross section (RCS) prediction [4–6]. The essence of computational electromagnetics is to take Maxwell’s equations, which are highly generalized by macroscopic electromagnetic theory, as the mathematical model, combine with the initial and boundary conditions of practical problems, and give the solution of electromagnetic problems. For simple objects, such as spheres and cylinders, the analytical solutions of electromagnetic problems can be deduced by Maxwell’s equations.

However, the objects in practical problems are often more complex, such as the helicopter. It is impossible to obtain the solution of electromagnetic problems for electrically large complex objects by analytical methods. Considering that the analytical methods can only solve simple problems with regular boundaries, a variety of numerical methods have been proposed to approximate the solution of the original problem, such as the moment method (MOM) [7, 8], the finite element method (FEM) [9], and finite-difference time-domain (FDTD) [10], among which the moment method is the most widely used. In 1968, R. F. Harrington introduced MOM into computational electromagnetics for the first time, which achieved high computational accuracy for electromagnetic problems and good adaptability to the 3D object with arbitrary shape [7]. After decades of development, MOM is still used as the benchmark algorithm for other numerical methods.

Several computational difficulties limit the application of classical numerical approaches. The first difficulty is that the size of the object in most problems is much larger than the wavelength, which is called the electrically large object, resulting in the excessive cost of hardware resources in these methods, especially in the military field. Therefore, these methods are usually deployed on cluster servers. Another dilemma is that even with cluster servers, classical numerical approaches can not produce results in a reasonable time.

With the increase of radar frequency, these shortcomings will become more and more obvious. Then, many asymptotic methods have been taken into account, such as geometrical optics (GO) [11], physical optics (PO) [12, 13], and method of equivalent currents (MEC) [14–16], which are based on the fact that the coupling effect between the facets of the object can be approximately ignored under the irradiation of high-frequency electromagnetic wave. Different methods correspond to different scattering mechanisms. Because of its ability to accurately simulate the multiple reflection mechanism, the shooting and bouncing rays (SBR) method has been one of the basic tools for over four decades when high-frequency RCS prediction of complex electrically large objects is of concern [17, 18]. It is a hybrid method of geometrical optics combined with physical optics. The GO method is used to simulate the multiple reflections of thousands of ray tubes between various parts of the object, and then the PO method is used to solve the far-field integral of each ray tube.

Therefore, the SBR method mainly includes three parts, which are ray path tracing, PO radiation integral, the superposition of the fields of each ray tube. Hence, the factors that affect the performance of the SBR method are mainly in two aspects, one is the ray path tracing, the other is the calculation of the far-field integral of millions of ray tubes. As soon as the ray path tracing is completed, the far-field computation for each ray tube is independent of each other, which means that it is easily accelerated by parallel techniques, such as multithreading or GPU acceleration. Therefore, the core of the SBR algorithm is to accurately and efficiently trace the enormous quantity of rays according to the geometrical features of complex objects.

To improve the computational efficiency of ray tracing, a variety of spatial acceleration structures have been developed to reduce the number of intersection tests in the process of intersection between each ray and all facets of the object, such as octree structure [19] and kd-tree structure [20, 21]. The traversal of these acceleration structures is usually recursively implemented, which is not supported on the GPU. Based on this fact, researchers have proposed a variety of kd-tree traversal algorithms adapted to GPU architecture [22–24]. Meanwhile, the new data structure based on GPU such as bounding volume hierarchies (BVHs) was proposed to speed up the construction and traversal of the scene, which reduced the complexity of ray tracing to *log*(*N*), where *N* is the number of tessellated triangle faces [25–27]. It also shows that the performance of BVHs on GPU is better than that of kd-tree.

While there are existing ray tracing algorithms on GPU, they are generally used in the field of computer vision. It is worth noting that they all regard GPU as a small cluster server, which only takes advantage of thousands of parallel-processing cores in a single GPU. Benefiting from the demand for high-performance graphics cards in the gaming field, NVIDIA released an RTX series of graphics cards with a new Turing architecture in 2018. RTX graphics card integrates multiple dedicated ray tracing processors, which can accelerate the propagation of light and sound in 3D environments at a speed of up to 10 Giga Rays per second. Therefore, it is promising to study the asymptotic method suitable for the Turing architecture to make full use of the built-in RT Cores.

In this paper, we present the acceleration of the SBR method based on OptiX [28] and normal vectors correction. Unlike other GPU-based acceleration algorithms, the method not only utilizes GPU parallel computing, but also uses RT Cores to accelerate the algorithm from the hardware level. Additionally, normal vectors correction is introduced in ray path tracing to obtain ray path more accurately and to improve the precision of scattered field radiated by ray tubes. Firstly, the basic principle of the SBR algorithm is introduced, and the key points affecting the performance of the algorithm are pointed out. Then, a detailed description of the accelerated algorithm is presented, followed by the simulation results and finally, conclusions are shown regarding the performance of our method.

## Materials and methods

### Brief overview of SBR

When the scatterer is illuminated by the electromagnetic wave, it will interact with the incident wave to generate a scattered field. The SBR algorithm is a hybrid method combining PO and GO, which can be divided into four stages. Electromagnetic propagation is first represented with the reflection, refraction, and divergence of optical rays. Millions of ray tubes are launched from the source to the scatterer, and the paths of each ray tube bouncing with the scatterer surfaces are traced according to Snell’s law. Next, the electromagnetic properties of magnitude, direction, and phase are then added on top of the ray traces to mimic the properties of waves. Then, the contribution of each ray tube to the scattered field is independently calculated. Finally, the final scattered field of the scatterer under the irradiation of the incident wave can be expressed as the superposition of individual contributions from all ray tubes.

**Fig 1:**
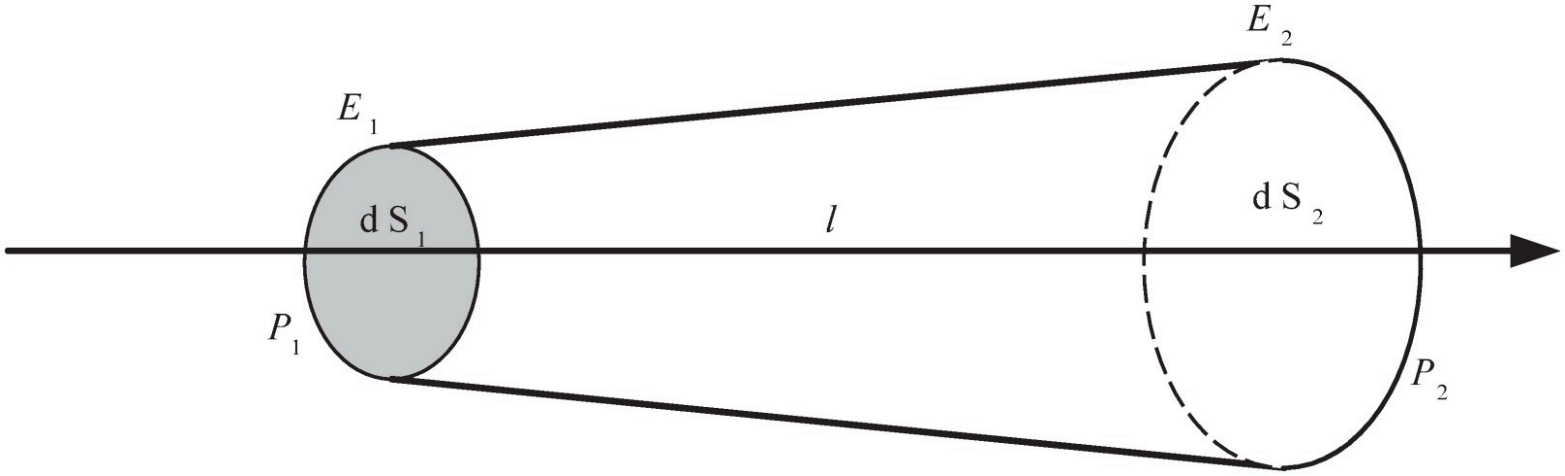
The propagation path of rays in a homogeneous medium.

Since the cross-sectional area at both ends of the ray tube may change to a certain extent when the ray tube intersects the next facet, it is necessary to correct the diffused electromagnetic wave energy. Fig 1 shows a schematic diagram of the propagation path of rays in a ray tube with a homogeneous medium, where *P*_1_ is the inflow end and *P*_2_ is the outflow end. Suppose that the field of each ray in any transverse cross-section of the ray tube is the same as that of the central ray. Given the incident electric field **E**_**1**_, the electric field for the center ray **E**_**2**_ at *P*_2_ is determined by the law of conservation of energy, as shown in Eq (1).

$$\begin{array}{c} {\left|E_{1}\right|}^{2}dS_{1}={\left|E_{2}\right|}^{2}dS_{2} \end{array}$$ (1)

where *dS*_1_ and *dS*_2_ represent the cross-sectional area of the ray tube at *P*_1_ and *P*_2_, respectively.

According to Eq (1), the change of the electric-field intensity along the direction of the ray tube can be obtained. Since the distance of the ray propagating is *l* m, the spatial phase delay is exp(−*jkl*). Therefore, the electric field at the outflow end *P*_2_ can be expressed as

$$\begin{array}{c} E_{2}=\sqrt{\frac{dS_{1}}{dS_{2}}}E_{1}\exp\left(-jkl\right) \end{array}$$ (2)

When considering multiple reflections, the electric field at each intersection of the ray tube can be solved recursively by Eq (2). At this point, the tracking of the electric field during the propagation of the ray tube can be completed.

The electric field in Eq (2) represents the incident electric field of the triangular facet where the ray tube and the object intersect. From Stratton-Chu equations [29], the scattered field of the ray tube in the far region is

$$\begin{array}{c} E^{s}\left(r\right)=\frac{jk_{0}e^{-jk_{0}r}}{4\pi r}\int_{S}[\hat{s}\times (\hat{s}\times (\hat{n}\times (\hat{i}\times E^{i}(r^{\prime }))))+\hat{s}\times (E^{i}(r^{\prime })\times \hat{n})]e^{jk_{0}\hat{s}\cdot r^{\prime }}ds \end{array}$$ (3)

where $\hat{i}$ is the propagation direction of the incident ray during the last reflection, $\hat{s}$ is the unit vector of the observation point, *r* is the distance between the observation point in the far region and the triangular facet, and **E**^*i*^(**r**′) is the incident electric field at the facet determined by Eq (2). Under the far-field assumption, *r* → ∞.

The total scattered field of the scatterer can be obtained by superposing the scattered fields of millions of ray tubes, *i.e*.

$$\begin{array}{c} \begin{array}{c} E_{T}^{s}\left(r\right)=\frac{jk_{0}e^{-jk_{0}r}}{4\pi r}\sum_{m=1}^{M}C_{m}\int_{\Delta S_{m}}e^{jk_{0}{r^{\prime }}_{m}•\left(\hat{s}-\hat{i}\right)}dS\\ C_{m}=[\hat{s}\times \hat{s}\times \left(\hat{n}\times \left(\hat{i}\times E^{i}\left({r^{\prime }}_{m}\right)\right)+\hat{s}\times \left(E^{i}\left({r^{\prime }}_{m}\right)\times \hat{n}\right)\right]e^{jk_{0}\hat{i}\cdot {r^{\prime }}_{m}} \end{array} \end{array}$$ (4)

where *M* represents the total number of ray tubes.

It is difficult to directly solve the surface integral in Eq (4). Therefore, to simplify the calculation of the scattered field, the Gordon algorithm can be used to convert the surface integral to the line integral on the three sides of the triangle [12].

### Real-time ray tracing based on OptiX

The principle of the SBR method shows that there are two time-consuming processes. The first time-consuming part is that millions of ray tubes are launched from the source to the scatterer, and the paths of each ray tube bouncing with the scatterer surfaces need to be traced. Suppose the 3D model of the scatterer is described by *N* flat triangles. In order to obtain accurate results, a considerable number of rays (*M*) are required to be shot to the scatterer. Evidently, the operational complexity of direct ray tracing is *O*(*MN*). For realistic objects, *N* is often more than one million, which leads to the unacceptable time of ray tracing. As both *N* and *M* can be large numbers, it is necessary to speed up the ray-triangle intersection algorithm.

OptiX is a general purpose ray tracing engine designed for NVIDIA GPUs to achieve optimal ray tracing performance. The core of OptiX is a domain-specific just-in-time compiler that generates custom ray tracing kernels by combining user-supplied programs for ray generation, material shading, object intersection, and scene traversal [28]. Its general programming interface enables it to be applied not only to graphic but also to non-graphic domains. Unlike other ray tracing engines, OptiX is intended for ray tracing applications that use NVIDIA CUDA technology, such as film and television visual effects. Since ray tracing is a prerequisite for the SBR method, this fact determines that OptiX can be applied to the high-frequency asymptotic methods.

**Fig 2:**
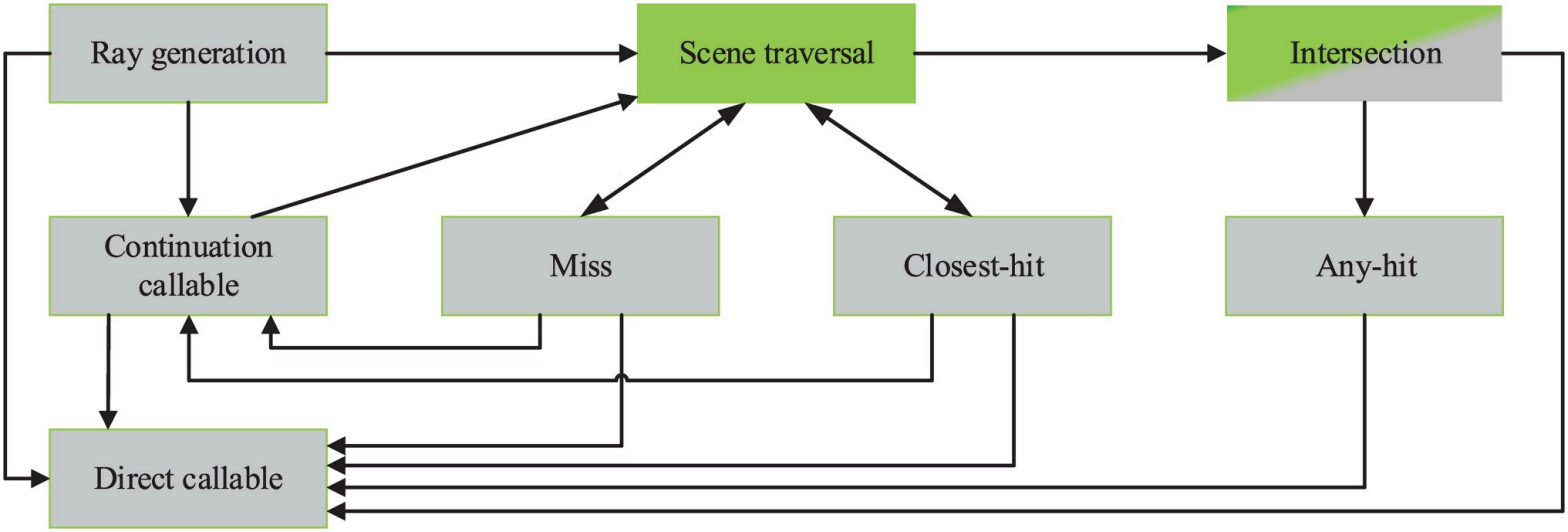
Relationship of OptiX programs. The green boxes represent fixed algorithms provided by the OptiX; the gray boxes represent user-specified programs.

There are eight types of user-defined ray interactions, called programs, which are interconnected by scene traversal. The relationship between these programs is shown in Fig 2. In addition to scene traversal, seven other programs and exception programs constitute the above eight programs. It is worth noting that the gray boxes in Fig 2 represent user-specified programs, which are not implemented in OptiX. The user is responsible for their specific implementation, such as ray generation, direct callable, and miss. Similar to a regular CUDA function call, direct callables are called immediately. Unlike direct callables, continuation callables are executed by the scheduler. Both of them allow for additional programmability within the standard set of NVIDIA OptiX programs. The ray generation program generally contains three parts, which are creating rays, starting scene traversal, and storing the results of ray tracing. When considering multiple reflections, the current incident direction, intersection point, and other relevant information will be used as the basis for creating the reflected ray. The intersection program implements ray-geometry intersection tests. When a traced ray finds the closest intersection point, the closest-hit will be called, such as for material shading. Miss program is executed when the ray does not intersect any geometry in the scene. Unlike closest-hit, any-hit will be called when a traced ray finds a new, potentially closest, intersection point. It does not provide the closest point of the intersection but only indicates that the ray intersects with geometry in the scene, and is usually used for shadow computation. If exceptions are enabled, the built-in or user-provided exception program may be called from any program or scene traversal when an exception occurs.

In OptiX, the geometric data of the scene are first built as acceleration structures on the GPU, which contains the 3D model of the scatterer as well as static or dynamic transformations. Then the ray generation program constructs the ray tube by specifying the source point, direction, and parameters. After that, the ray tube is ready for launch. For determining the state of ray propagation in the scene, OptiX searches a graph of nodes composed of acceleration structures and transformations. If the ray hits the surface, the closest-hit program will be triggered, which can provide hit point coordinate, hit surface element, and other relevant information. If the ray has no intersection with the scene, the miss program will be triggered, which is generally used to mark the background color or indicate that the ray has no intersection point.

To speed up the search for the intersection of rays with the geometric data in the scene, OptiX uses the BVH model to build the acceleration structures. Although it is difficult to achieve efficient construction of accelerated structures and efficient traversal of a scene graph, they are built-in functions and done automatically by the OptiX. Thus, the implementation of programs such as ray generation, closest-hit, and miss is of concern.

Unlike the graphic domain, rays are not produced by point source of light. In the far-field condition, the incident electromagnetic wave can be approximated as a plane wave. Therefore, these rays are parallel to each other, which means that they have different starting points but the same direction, as shown in Eq (5).

$$\begin{array}{c} P=P_{0}-c\hat{k} \end{array}$$ (5)

where *P*_0_ is the reference point of the virtual aperture, *c* is a positive constant, and $\hat{k}$ is the unit vector of the incident direction.

It is worth noting that the ray generator not only emits rays but also invokes the scene traversal algorithm to determine the state of rays. After the execution of the closest-hit program is completed, the hit point coordinate and the corresponding surface element will be returned to the ray generation. Therefore, the reflected ray $\hat{s}$ can be obtained simply through the normal vector of the triangle facet $\hat{n}$ and the incident direction $\hat{i}$, which can be expressed as

$$\begin{array}{c} \hat{s}=\frac{s}{s} \end{array}$$ (6)

where

$$\left\{ \begin{array}{c} s=\hat{i}+2\cos\theta \hat{n}\\ \cos\theta =-\hat{i}\cdot \hat{n} \end{array}\right.$$

and the source point is the hit point.

Therefore, the new ray $\hat{s}$ will be generated and launched in the ray generation program. This process is performed recursively to obtain the path of the ray bouncing with the scatterer surfaces. In the miss program, the ray will be terminated, which means that the life cycle of the ray ends.

To increase the speed of calculation, there are three main approaches in OptiX, parallelism, BVH, and RT Cores. Parallelism is based on the fact that a GPU has a massively parallel array of integer and floating-point processors, as well as the utilization of multiple GPU cores. OptiX assigns millions of rays to multiple threads and performs ray tracing at the same time to achieve very high levels of parallelism. Unlike the kd-tree spatial splits, the BVH divides the space through all information of the objects so as to achieve efficient scene segmentation. After spatial splits, objects are surrounded by slightly overlapping bounding boxes. At this point, the intersection operation of rays and objects will be converted into that of rays and bounding boxes. If the ray does not touch the bounding box, it will not intersect the objects inside the bounding box. This will speed up the intersection test between rays and facets of objects. The third approach is the most important, which is the core of OptiX to achieve the state of the art ray tracing. By invoking the built-in RT Cores of hardware, OptiX can greatly reduce the time consumption of ray tracing, thereby realizing real-time ray tracing.

### Normal vectors correction

In path tracing, the direction of the reflected ray is determined by the normal vector of the hit facet and the direction of the incidence ray, as shown in Eq (6). However, real objects often contain a large number of curved surfaces, which for convenience are often modeled using triangular facets. Although there were SBR methods based on NURBS surfaces [30], the performance loss is greater than that of triangular facets. Therefore, triangular facets are often used in practice.

The use of triangular facets implies that the reflection of rays at locally flat surfaces. If the reflected ray interacts with the object during subsequent propagation, neglect of the surface curvature might significantly affect the simulation results. Therefore, to accurately predict the RCS of any triangular facetted objects, the curvature of the surface of the objects must be taken into account. This means that for an arbitrary curved surface represented by triangular facets, the principal radii of curvature *R*_1_ and *R*_2_, the corresponding directions ${\hat{e}}_{1}$ and ${\hat{e}}_{2}$, the hit point *P*, and the normal $\hat{n}$ at this point need to be calculated during ray tracing. If the curvature is not considered, which is a special case of normal vectors correction, the normal $\hat{n}$ will be the normal vector of the triangular facet, i.e. *R*_1_ = *R*_2_ = ∞.

**Fig 3:**
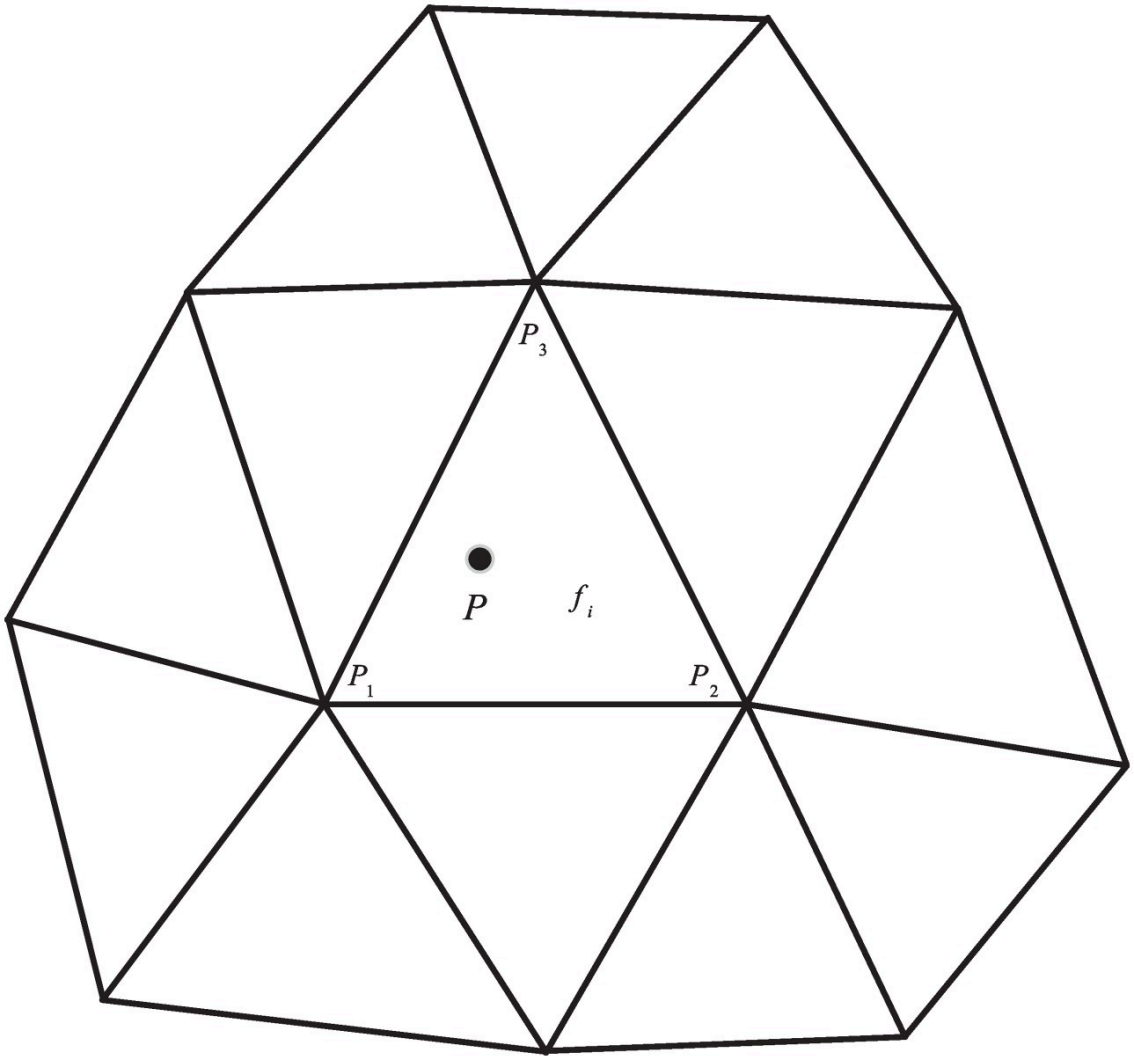
The neighboring triangles of hit point *P*.

Suppose the hit point *P* lies on a triangle *f*_*i*_ with points *P*_1_, *P*_2_, and *P*_3_. It is easy enough to guarantee that the three vertices are located on the real curved surface. To calculate the curvature information of point *P*, the neighboring triangles of *f*_*i*_, which share vertex with *f*_*i*_, need to be analyzed, as shown in Fig 3. The number of the neighboring triangles is not fixed, which is related to the local geometry. But, it contains at least six non-repeated vertices. In the local region of *P*, the curved surface of the object can be approximated as a quadratic polynomial surface, which can be expressed as

$$\begin{array}{c} w\left(u,v\right)=a_{2,0}u^{2}+a_{0,2}v^{2}+a_{1,1}uv+a_{1,0}u+a_{0,1}v+a_{0,0} \end{array}$$ (7)

where *a*_*i*,*j*_ is the undetermined coefficient. The coordinate system (*u*, *v*, *w*) forms the local coordinate system of the triangle *f*_*i*_, where the uOv-plane is the plane of the triangle. To avoid ambiguity, the origin of the local coordinate system is located at the center of mass of the triangle *f*_*i*_.

For the six undetermined coefficients in Eq (7), at least six vertices are needed for the least square fitting, which is easy to satisfy. Thus, the system of equations with undetermined coefficients can be expressed as

$$\begin{array}{c} w_{j}=a_{2,0}u_{j}^{2}+a_{0,2}v_{j}^{2}+a_{1,1}u_{j}v_{j}+a_{1,0}u_{j}+a_{0,1}v_{j}+a_{0,0},\;\;\;\;j=1,2,3,\ldots ,m \end{array}$$ (8)

where *m* represents the number of vertices.

By vectorizing Eq (8), it can be simplified to

$$\begin{array}{c} \begin{array}{c} Aa=w\\ A=[\begin{array}{cccccc} u_{1}^{2} & v_{1}^{2} & u_{1}v_{1} & u_{1} & v_{1} & 1\\ u_{2}^{2} & v_{2}^{2} & u_{2}v_{2} & u_{2} & v_{2} & 1\\ \vdots & \vdots & \vdots & \vdots & \vdots & \vdots \\ u_{m}^{2} & v_{m}^{2} & u_{m}v_{m} & u_{m} & v_{m} & 1 \end{array}],a=[\begin{array}{c} a_{2,0}\\ a_{0,2}\\ a_{1,1}\\ a_{1,0}\\ a_{0,1}\\ a_{0,0} \end{array}],w=[\begin{array}{c} w_{1}\\ w_{2}\\ \vdots \\ w_{m} \end{array}] \end{array} \end{array}$$ (9)

According to the method of least squares, the solution of the system of Eq (9) can be expressed as

$$\begin{array}{c} a={\left(A^{T}A\right)}^{-1}A^{T}w \end{array}$$ (10)

Consequently, the local curved surface at the hit point *P* can be approximately expressed by Eqs (7) and (10). The relevant curvature information can be accurately obtained by differential operation. Thus, the normal vector at point *P* can be corrected according to the curvature to improve the accuracy of ray tracing.

### Acceleration of far-field integral

Since ray tracing based on OptiX has achieved an optimal level, the second time-consuming part of SBR method is the calculation of the far-field integral of millions of ray tubes. Because the scattering contribution of each ray tube is independent of each other, the calculation of far-field integral can be accelerated by multithreading. If the multi-threading technology of the CPU is used to accelerate the calculation of the far-field integral, the result of the ray tracing needs to be transmitted from the GPU back to the CPU, which involves the transmission of data between heterogeneous devices. Because this kind of data transmission uses the PCI-E bus, the transmission rate is slow, which will greatly affect the performance of the SBR algorithm.

**Fig 4:**
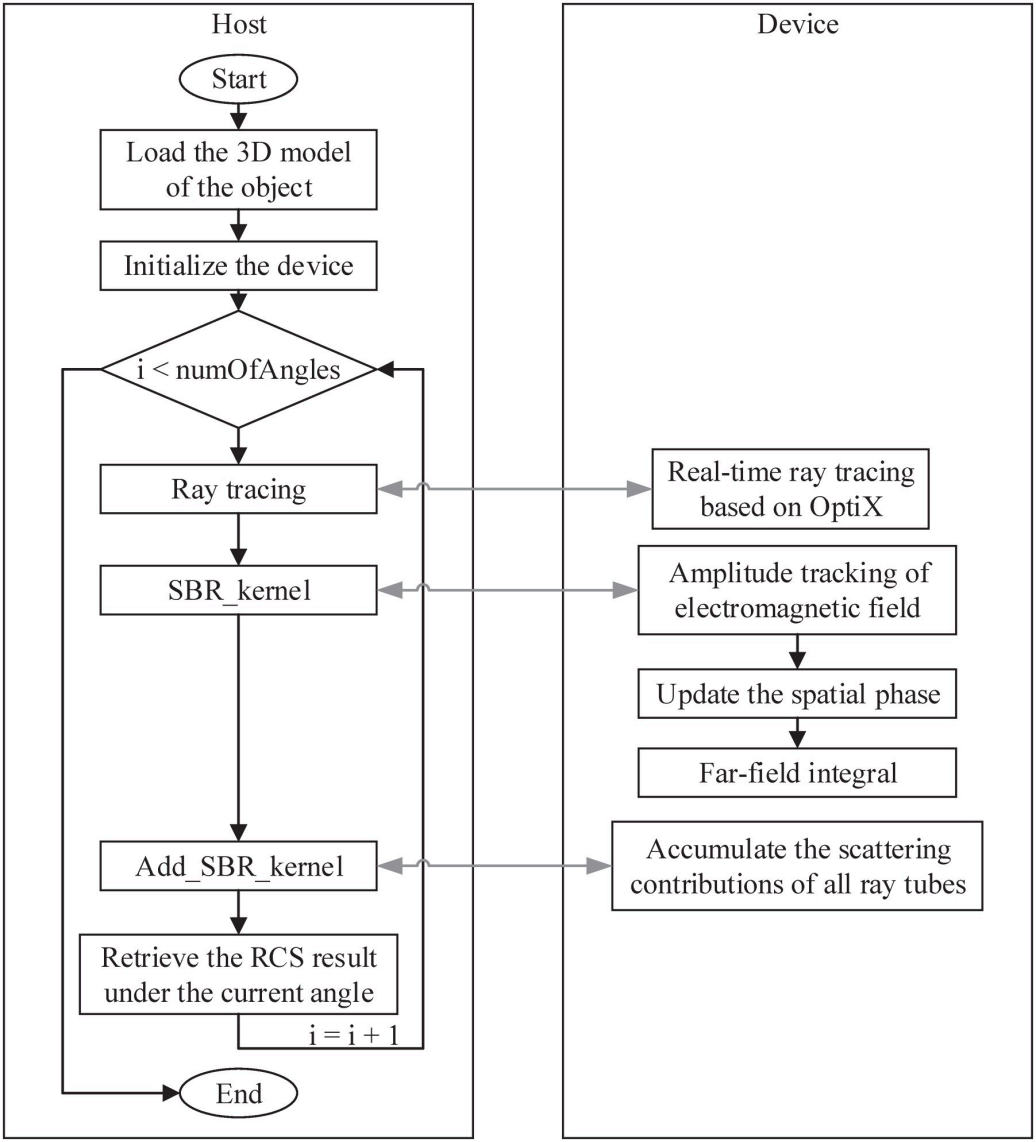
Flowchart of the proposed method.

Fortunately, OptiX is intended for ray tracing applications that use NVIDIA CUDA technology, which can be seamlessly integrated with CUDA to maximize the use of GPU resources while avoiding additional data transmission. Considering that millions of ray tubes are emitted uniformly from the virtual aperture, therefore, in the CUDA program, the number of blocks and threads per block is two-dimensional, and each thread corresponds to a ray tube on the aperture. Therefore, the performance bottleneck is solved through the concurrent execution of thousands of threads. Fig 4 shows the flowchart of the acceleration of shooting and bouncing ray method based on OptiX and normal vectors correction.

It can be seen from Fig 4 that the proposed method consists of two parts. One part is the CPU codes that control the process of the whole program, and the other part is the GPU part that does the parallel work. Since the GPU cannot read files on the hard disk, the CPU is responsible for reading the geometry model file of the object into the memory. Then, the CPU starts to allocate video memory and copy the data from the memory to the video memory. For RCS prediction, the RCS within a certain angle range is generally calculated. First of all, for the starting angle, the real-time ray tracing algorithm based on Optix and normal vectors correction introduced above is used to track the path of millions of ray tubes. On this basis, the kernel function “SBR_kernel” is called to calculate the scattering contributions of these ray tubes in parallel. Although the superposition of the scattering contributions of all ray tubes can be performed on the CPU, to avoid the transmission of a large amount of data between heterogeneous devices, the proposed method calls another kernel function “Add_SBR_kernel” to complete this summation process. Finally, the RCS result under the current angle is sent back to the CPU. This process will continue until the calculation of RCS at all angles is completed.

Therefore, far-field radiation integrals of millions of ray tubes can be accelerated using CUDA. Data transmission between heterogeneous devices only exists in the beginning and end stages, and the efficiency of the algorithm will be greatly improved. The SBR method based on OptiX and normal vectors correction is completely accelerated on the RTX GPU. And the RT Cores greatly speed up the efficiency of the SBR method, so that the personal laptop can predict the RCS of complex electrically large objects.

## Results

To verify the correctness and efficiency of the SBR method based on OptiX and normal vectors correction, a CPU-based version, called the SBR method based on kd-tree, is also implemented for comparison. Three-dimensional models of different objects have been established, and several numerical experiments have been carried out. These experiments are run atop Windows 10 on a personal laptop. The GPU hardware used in this paper is GeForce RTX 2070 (Notebooks), with 8 GB of memory. The CPU hardware used in this paper is Intel i7–9750H. Both methods are implemented in C++, and the development environment is Visual Studio Community 2017.

**Fig 5:**
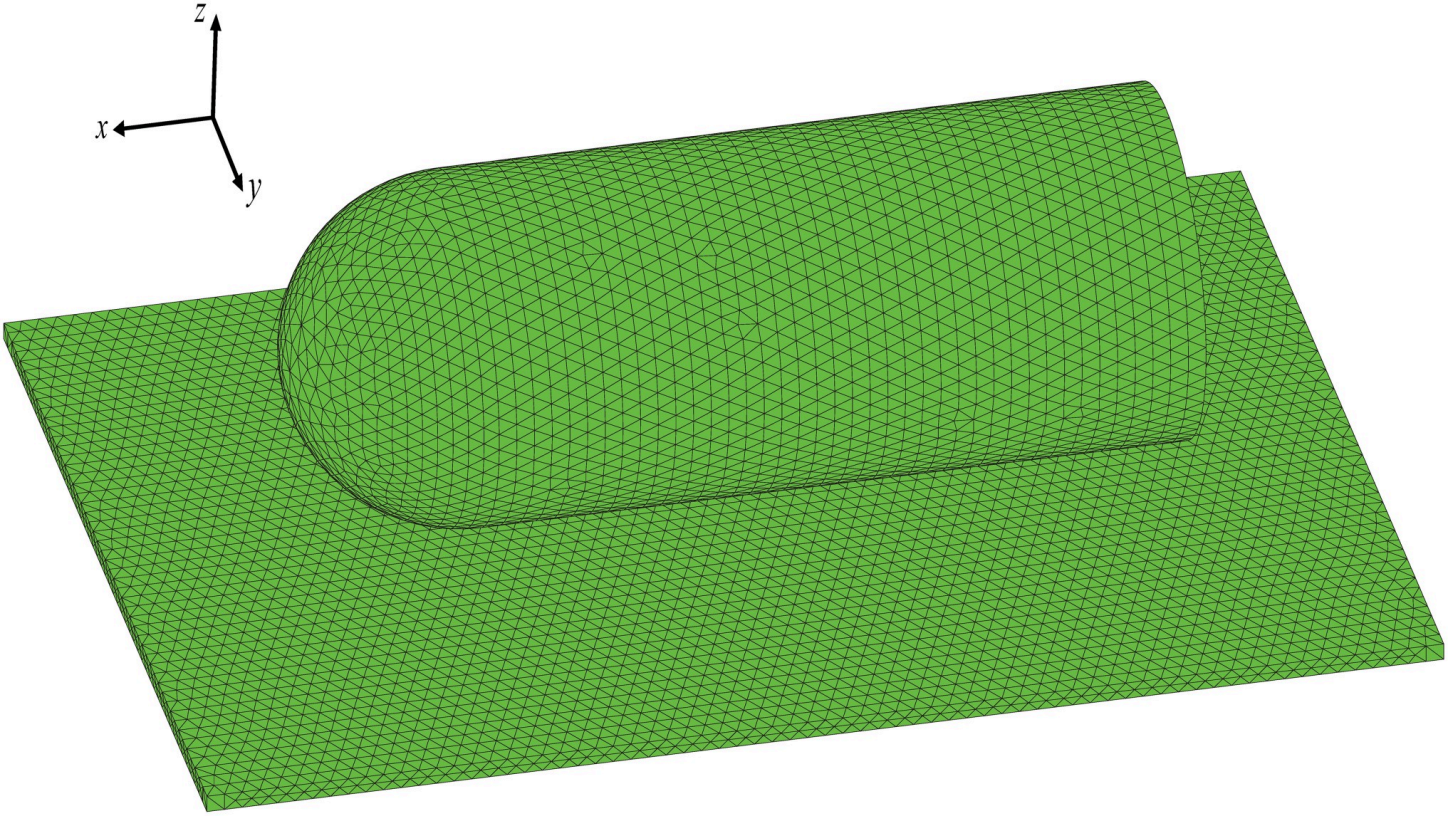
Test case of multi-object model.

For correctness verification, a simple multi-object model is created. The scenario consists of a metallic cylinder with a half-sphere on one end, both floating on a metallic cuboid, as shown in Fig 5. The length, width, and height of the cuboid are 1.4 m, 1 m, and 2 cm, respectively. For the sphere-cylindrical combination, the length of the cylinder is 0.8 m, and the radius of it and the sphere is 0.2 m. The gap between the combination and the cuboid is 2 cm. Suppose the operating frequency of the radar is 10 GHz. To guarantee convergence of results with respect to the surface representation, the objects are dissected by a mesh size of 1 cm, resulting in 88, 776 triangular facets. The three-dimensional geometric model after tessellation is shown in S1 Text.

Theoretically, RTX series graphics cards promise to deliver about 10 Giga Rays of ray tracing performance, a new performance metric that tracks the card’s ray tracing operations per second, thus realizing real-time ray tracing. The ray tracing performance of different graphics cards is shown in Table 1. It indicates that RTX 2070 graphics card can achieve 6 Giga Rays/s.

**Table 1:** The ray tracing performance of different graphics cards.

Graphics card model	Ray tracing performance
RTX 8000^①^	11 Giga Rays/s
RTX 2080Ti^②^	10 Giga Rays/s
RTX 2080 Super^③^	8 Giga Rays/s
RTX 2080^④^	8 Giga Rays/s
RTX 2070 Super^⑤^	7 Giga Rays/s
RTX 2070^⑥^	6 Giga Rays/s

^①^ https://www.nvidia.com/en-us/design-visualization/quadro/rtx-8000/

^②^ https://www.nvidia.com/en-us/geforce/graphics-cards/rtx-2080-ti/

^③^ https://www.nvidia.com/en-us/geforce/graphics-cards/rtx-2080-super/

^④^ https://www.nvidia.com/en-us/geforce/graphics-cards/rtx-2080/

^⑤^ https://www.nvidia.com/en-us/geforce/graphics-cards/rtx-2070-super/

^⑥^ https://www.nvidia.com/geforce/graphics-cards/rtx-2070/

**Fig 6:**
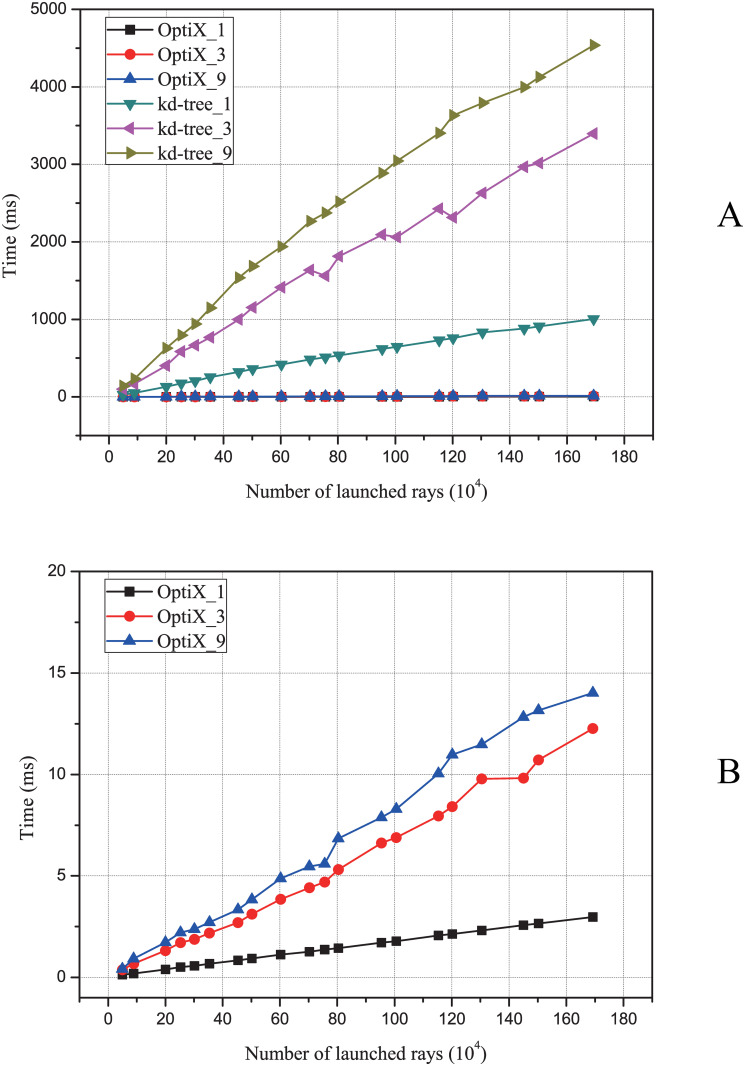
The ray tracing performance of different algorithms. (Panel A), the relationship between the simulation time and the number of rays under different number of reflections for the ray tracing method based on kd-tree and the ray tracing method based on OptiX. (Panel B) the performance of the OptiX-based method under different number of reflections.

For verification of the performance of ray tracing in electromagnetic simulation calculations, a ray tracing algorithm based on OptiX is studied and compared with the traditional method based on kd-tree. For this scenario, the ray tracing performance of different algorithms is compared, so different numbers of rays are launched from the source to the scatterer, which may lead to inaccurate results of the SBR method. Fig 6(A) shows the relationship between the time consumed by the ray tracing algorithm and the number of rays under the different number of reflections for the two methods. The number *i* in kd-tree_*i* and OptiX_*i* represents the number of reflections. It can be seen that the simulation time is approximately proportional to the number of rays. The more times the ray bounces with the target surface, the longer it takes to complete the ray tracing. Moreover, the ray tracing algorithm based on kd-tree is significantly slower than the ray tracing algorithm based on OptiX. Due to the large time-consuming difference between the two methods, Fig 6(A) cannot show the details of the performance of the ray tracing algorithm based on OptiX, so Fig 6(B) only shows the performance of the OptiX-based method under the different number of reflections. It can be seen that even with 9 reflections, the time spent on tracing 1.7 million rays is less than 15 milliseconds. For one reflection, it takes only 2.97 milliseconds. It can be calculated that the ray tracing algorithm based on OptiX can complete 0.57 Giga rays tracing in 1 second, which is 338 times faster than the ray tracing method based on kd-tree. Although there is a gap with the peak performance, this is because: 1) the GPU hardware used in this paper is a mobile graphics card for notebooks; 2) the GPU hardware also needs to be responsible for tasks such as display; 3) the results are related to the object.

**Fig 7:**
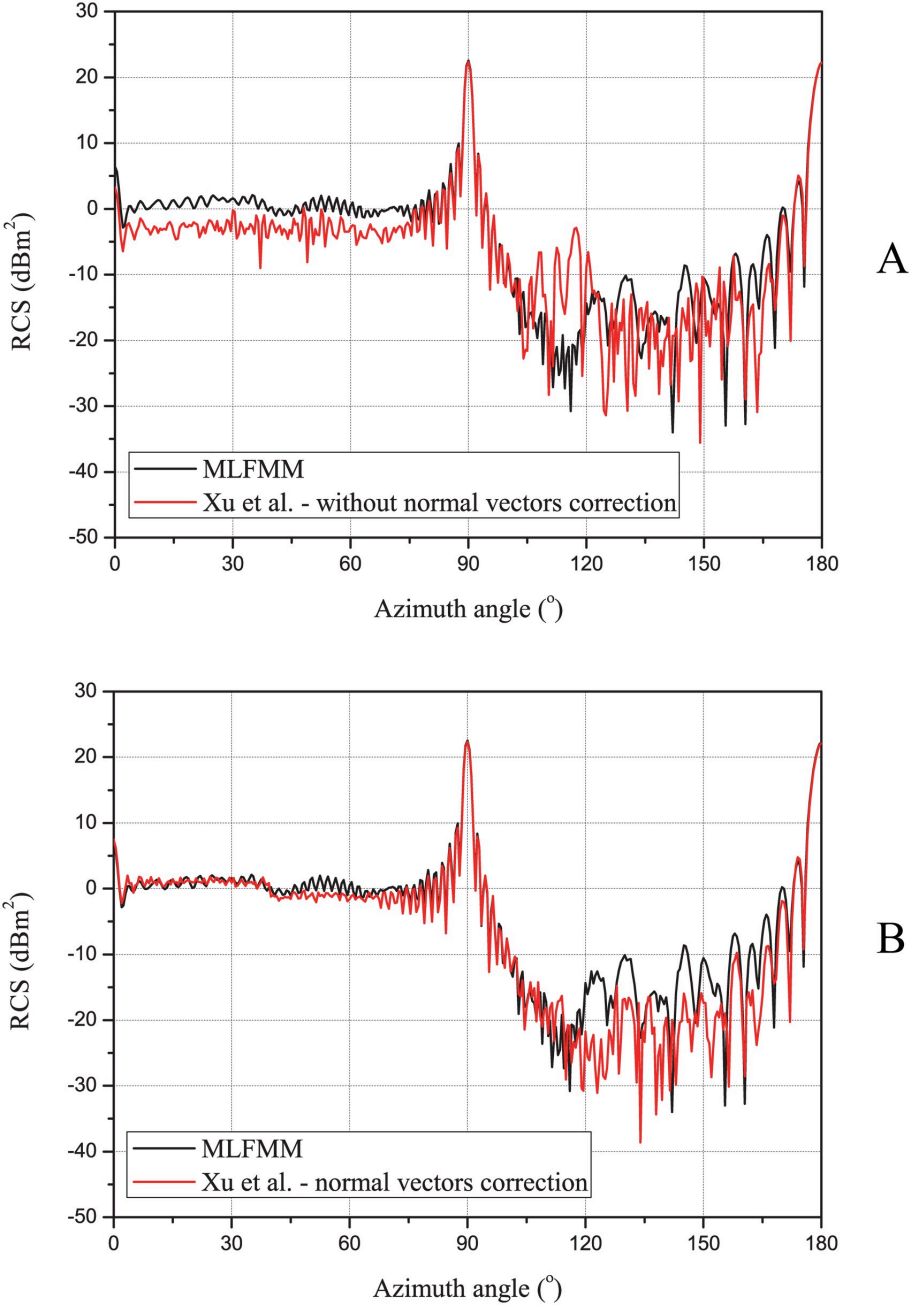
The RCS results of our method and MLFMM algorithm. (Panel A), our method without normal vectors correction. (Panel B) our method with normal vectors correction.

The reflected direction of the rays, using the facetted surface without normal vectors correction, will be deviated, which will adversely impact the object’s RCS. Therefore, the RCS results can be used to determine whether the normal vectors need to be corrected. Unlike the ray tracing test, scattering simulations are performed for HH-polarization (horizontal transmit and horizontal receive) at the frequency *f* = 10 GHz with an elevation angle *θ* = 35°. The azimuth angle ranges from 0° to 180° with an interval of 0.5°. The geometric model of the object is shown in Fig 5. The number of rays in this scenario is set to 1 million per aspect angle, and the maximum number of reflections of rays is set to 9. Fig 7 shows the RCS results for the test scenario, using the facetted surface without normal vectors correction (Fig 7A) and the facetted surface with normal vectors correction (Fig 7B), respectively, and the comparison with the exact solution based on the multilevel fast multipole method (MLFMM). It should be noted that the RCS results include the edge diffraction contribution calculated by the MEC method. Comparing the results with the exact solution obtained by the MLFMM algorithm, it can be seen that the facetted surface representation without normal vectors correction causes significant errors in RCS prediction, especially in the angular range from 0° to 80° and 105° to 125°. The former approximately underestimated 4 dB, while the latter approximately overestimated 15 dB. The results of the normal vectors correction are depicted in Fig 7(B). Good agreement in comparison with the exact solution is observed, especially in the angular range from 0° to 80°. Therefore, by using the normal vectors correction, the facetted surface can not only represent the object geometries efficiently but also can be used to accurately predict the RCS data.

On the other hand, the precise influence of normal vectors correction on the ray path can be analyzed by tracing a single ray. For the convenience of calculating the exact coordinates of the intersection point between the ray and the object surface, the azimuth angle of the incident ray is taken as 90 degrees. At this time, the propagation path of the ray is located in the YOZ-plane. The coordinates of each hit point under multiple reflections are calculated and compared with the analytical solution, as shown in Table 2. The error of the ray tracing without normal vectors correction is very large, causing obvious errors in the RCS prediction, which is consistent with the simulation results. However, after normal vectors correction, the ray tracing results are in good agreement with the analytical solution. Therefore, it is necessary to introduce normal vectors correction in the SBR method.

**Table 2:** The coordinates of each hit point under multiple reflections.

Reflection	Analytical solution	Without normal vectors correction	Normal vectors correction
1	(−257.78, 165.24, 107.33)	(−257.78, 164.86, 106.79)	(−257.78, 164.86, 106.79)
2	(−257.78, 93.99, 0)	(−257.82, 99.04, 0)	(−257.78, 92.95, 0)
3	(−257.78, 71.85, 33.35)	(−257.84, 77.14, 35.53)	(−257.78, 70.75, 32.97)
4	(−257.78, 76.85, 0)	(−258.27, 86.01, 0)	(−257.78, 75.08, 0)
5	(−257.78, 82.53, 37.82)	(−258.82, 97.31, 45.32)	(−257.78, 79.90, 36.69)
6	(−257.78, 141.38, 0)	(−261.67, 228.58, 0)	(−257.78, 131.51, 0)

For comparing the efficiency of our method and the traditional SBR algorithm based on kd-tree, the scattering simulations of different objects are performed for HH-polarization at the frequency *f* = 10 GHz. In these experiments, except for the different objects, other calculation conditions are the same, resulting in different numbers of facets. Table 3 shows the calculating time of monostatic RCS simulations of different objects at *f* = 10 GHz. These objects include elementary scatterers, different types of multi-rotor Unmanned Aerial Vehicles (UAVs), and fixed-wing UAVs. The larger the size of the object, the greater the number of facets after its tessellation. It is worth noting that the unit of calculation time in the traditional kd-tree-based method and our method is hours and minutes respectively. When the number of facets exceeds 2 million, the former takes nearly two days to complete the RCS prediction, while our method can be completed in less than an hour. It can be seen that our method improves the performance by about 60 times compared with the traditional method. Although the number of facets is small, the acceleration is relatively low, which can be explained that the calculation time cannot conceal the data transmission between the CPU and the GPU. This situation will improve a lot when the number of facets is large.

**Table 3:** The calculating time of monostatic RCS simulations of different objects at *f* = 10 GHz.

Number of vertices	Number of facets	Calculating time		Speedup
		Traditional kd-tree-based method (h)	Our method (min)	
13,240	26,476	0.62	1.46	25.48
26,489	52,970	1.62	1.67	58.20
31,687	63,370	2.03	1.98	61.52
38,613	77,222	2.57	2.58	59.77
39,727	79,442	2.81	2.58	65.35
77,347	154,686	5.93	5.80	61.34
164,596	328,829	10.36	9.77	63.62
287,169	573,514	17.26	17.01	60.88
1,012,157	2,021,310	49.04	47.36	62.13

## Conclusion

A new efficient SBR algorithm based on OptiX and normal vectors correction is presented in this paper. For path tracing, the algorithm uses the built-in RT Cores of the RTX series graphics cards to accelerate processing, so the intersection of the ray and the object can be efficiently implemented. Moreover, for the calculation of the far-field integration of millions of ray tubes in the SBR method, the algorithm combined with CUDA acceleration technology makes full use of the computing resources of the graphics card and is seamlessly integrated with the ray tracing, which greatly reduces the data transmission time between the CPU and GPU heterogeneous devices and improves computational efficiency. Considering the model error introduced when the facetted surface represents the curved surface, the algorithm corrects the normal vector during path tracing, which significantly improves the accuracy of path tracing, and can accurately obtain the RCS data. Simulation results show that our method can efficiently and accurately predict the RCS of arbitrary facetted geometries, which is about 60 times faster than the traditional method and can be done with a personal laptop.

## Supporting information

S1 Text (TXT)
